# Signal transduction mechanisms involved in S100A4-induced activation of the transcription factor NF-κB

**DOI:** 10.1186/1471-2407-10-241

**Published:** 2010-05-28

**Authors:** Ida Grotterød, Gunhild M Mælandsmo, Kjetil Boye

**Affiliations:** 1 Department of Tumor Biology, Institute for Cancer Research, The Norwegian Radium Hospital, Oslo University Hospital, Montebello, 0310 Oslo, Norway; 2 Department of Pharmacy, Faculty of Health Sciences, University of Tromsø, 9037 Tromsø, Norway; 3 Department of Oncology, The Norwegian Radium Hospital, Oslo University Hospital, Montebello, 0310 Oslo, Norway

## Abstract

**Background:**

The metastasis-promoting protein S100A4 activates the transcription factor NF-κB through the classical NF-κB activation pathway. The upstream signal transduction mechanisms leading to increased NF-κB activity are, however, incompletely characterized.

**Methods:**

The human osteosarcoma cell line II-11b was stimulated with recombinant S100A4 in the presence or absence of inhibitors of common signal transduction pathways, and NF-κB activity was examined using a luciferase-based reporter assay and phosphorylation of IκBα. mRNA expression was analyzed by real-time RT-PCR, protein expression was examined by Western blotting and IKK activity was measured using an in vitro kinase assay. The role of upstream kinases and the cell surface receptor RAGE was investigated by overexpression of dominant negative proteins and by siRNA transfection.

**Results:**

The Ser/Thr kinase inhibitors H-7 and staurosporine inhibited S100A4-induced IκBα phosphorylation and subsequent NF-κB activation. The protein tyrosine kinase inhibitor genistein and the phospholipase C inhibitor compound 48/80 had a partial inhibitory effect on IκBα phosphorylation, whereas inhibitors of protein kinase C, G-protein coupled receptors and PI 3-kinases had no effect on the level of phosphorylation. Interestingly, S100A4 treatment induced activating phosphorylations of IKKα/β, but neither H-7 nor staurosporine was able to significantly inhibit IKK activation. Dominant negative MEKK1 or NIK did not inhibit S100A4-induced NF-κB activity, and S100A4 stimulation did not influence AKT phosphorylation. Furthermore, diminished expression of the putative S100 protein receptor RAGE did not affect the observed phosphorylation of IκBα.

**Conclusions:**

S100A4 activates NF-κB by inducing phosphorylation of IKKα/β, leading to increased IκBα phosphorylation. The Ser/Thr kinase inhibitors H-7 and staurosporine attenuated S100A4-induced NF-κB activation and inhibited IKK-mediated phosphorylation of IκBα. S100A4-induced NF-κB activation was independent of the putative S100 protein receptor RAGE and the Ser/Thr kinases MEKK1, NIK and AKT. These findings lead to increased understanding of S100A4 signaling, which may contribute to the identification of novel targets for anti-metastatic therapy.

## Background

The metastasis-promoting protein S100A4 belongs to the S100 family of structurally related calcium binding proteins [1,2]. The S100 proteins are expressed in a cell and tissue specific manner and are involved in a variety of cellular processes, such as cell cycle regulation, cell growth, differentiation and motility [3]. The protein level of S100A4 is elevated in several human cancers [1,2], and expression of the protein is correlated with poor prognosis in several malignancies, including breast and colorectal cancer [4,5]. Similar to other S100 proteins, S100A4 possesses both intracellular and extracellular functions. When applied extracellularly, S100A4 is able to promote metastasis, stimulate angiogenesis, induce cell motility and increase expression of matrix metalloproteinases [6-10]. Even though many of the biological effects are described, the mechanisms by which S100A4 exerts these effects are incompletely understood.

In a previous study we demonstrated increased NF-κB activity and phosphorylation of JNK (c-Jun N-terminal kinase) upon stimulation of II-11b cells with extracellular S100A4 [11]. S100A4-induced activation of NF-κB, ERK1/2 (extracellular signal-regulated kinase 1/2), p38 MAP kinase and JNK have also been demonstrated in other cell systems [6,10-13]. However, the connection between these kinases and NF-κB is not known, and the upstream mechanisms leading to S100A4-induced NF-κB activation have not been established. Receptor for Advanced Glycation End products (RAGE) has been suggested as a putative receptor for several S100 proteins [14]. RAGE-dependent activation of NF-κB and subsequent enhanced MMP-13 expression was observed in chondrocytes upon stimulation with S100A4 [10], but RAGE-independent effects have also been described [15]. Through interaction with annexin II S100A4 was able to induce angiogenesis [8], and in neurons heparan sulfate proteoglycans were necessary for S100A4-induced neurite extension [15]. Most likely, the protein also acts through so far unidentified mechanisms, and interaction with different receptors may explain the various biological effects of extracellular S100A4.

The heterodimeric transcription factor NF-κB is a central player in cancer development and progression. Schematically, NF-κB can be activated through either the classical or the alternative pathway. In the classical activation pathway NF-κB dimers are retained in the cytoplasm by binding a class of inhibitor proteins, called IκBs. Upon activation, the IKK (IκB kinase) complex phosphorylates IκBs and thereby targets the latter for proteasome-mediated degradation. NF-κB dimers can then translocate to the nucleus where they bind DNA and regulate transcription [16].

Here, we demonstrate that extracellular S100A4 stimulates NF-κB activity by inducing phosphorylation of the IKK complex and subsequent IKK-mediated phosphorylation of IκBα. The Ser/Thr kinase inhibitors H-7 and staurosporine reduced S100A4-induced IκBα phosphorylation and NF-κB activation, whereas inhibitors of other common signaling pathways had a minor or no effect. The Ser/Thr kinases MEKK1 (MEK Kinase 1), NIK (NF-κB Inducing Kinase) and AKT (protein kinase B), and the putative S100A4 receptor RAGE, were not involved in S100A4-induced NF-κB activation in the cell system investigated.

## Methods

### Materials

Mouse recombinant His-S100A4 was produced as previously described by Haugen et al [17]. Suramin, U-73122, genistein, AG 18, H-7 and staurosporine were purchased from Calbiochem (Darmstadt, Germany), GDPβ (guanosine 5'-[β-thio]diphosphate trilithium salt), compound 48/80 and GF 109203X were obtained from Sigma-Aldrich (St. Louis, MO), and LY294002 was purchased from Cell Signaling Technology (Beverly, MA).

### Cell culture and treatment

The human osteosarcoma cell lines KPDX, the in-house anti-S100A4 ribozyme transfected osteosarcoma cell line II-11b and its parent cell line OHS have been described previously [18-20]. The osteosarcoma cell line U2OS and the colorectal cancer cell lines HCT 116 and SW620 were obtained from ATCC (Rockville, MD). Cells were cultivated in RPMI-1640 (BioWhittaker, Verviers, Belgium) containing 8.5% fetal bovine serum (FBS; Biochrome KG, Berlin, Germany), 20 mM Hepes buffer (Lonza, Verviers, Belgium), and 2 mM Glutamax (GIBCO BRL, Life Technologies, Paisley, UK). Human calvarial osteoblasts (lot number 3417) were obtained from ScienCell Research Laboratories (Carlsbad, CA) and cultivated in poly-L-lysine coated flasks in Osteoblast Medium with Osteoblast Growth Supplement (ScienCell Research Laboratories, Carlsbad, CA). Subconfluent cultures were trypsinized and seeded at 6 × 10^4 ^cells/cm^2 ^unless otherwise stated. After overnight incubation, cell culture medium was replaced with fresh medium in the presence or absence of signal transduction inhibitors as indicated. The cells were further incubated for 30 minutes or one hour prior to addition of 2 μM S100A4, and harvested at the indicated time points. Relevant solute controls were included in all experiments

### Western blot analysis

Protein lysates were prepared as previously described [17]. Protease and phosphatase inhibitors were added to the lysis buffer just before use (10 μg/ml each of leupeptin, pepstatin and aprotinin, 1 mM PMSF, 5 mM NaF, 20 mM β-glycerophosphate and 0.5 mM sodium orthovanadate). Western blotting was performed as described previously [21], with the exception that protein lysates were separated on 4-12% NuPAGE^® ^Novex Bis-Tris Gels (Invitrogen, Carlsbad, CA) and that 5% non-fat dry milk was used in the blocking solution (10% for α-tubulin). Primary antibodies were diluted in 5% non-fat dry milk or BSA in Tris-buffered saline (TBS) containing the below noted percentages of Tween 20. Anti-phospho-IκBα (Ser32/36; 1:2000; 0.05% Tween 20; #9246), anti IκBα (1:1000; 0.1% Tween 20; #9242), anti-phospho-IKKα/β (Ser176/180 for IKKα and Ser177/181 for IKKβ; 1:1000; 0.1% Tween 20; #2687) and anti-phospho-AKT (Ser473; 1:500; 0.1% Tween 20; #9271) were obtained from Cell Signaling Technology (Beverly, MA). Anti-RAGE (1:500; 0.05% Tween 20; sc-80652) was obtained from Santa Cruz Biotechnology (Santa Cruz, CA), anti-IKKα (1:1000; 0.1% Tween 20; AF3768) from R&D systems (Minneapolis, MN), and anti-α-tubulin (1:1000; 0.25% Tween 20; CP06) from Calbiochem (Darmstadt, Germany). Signals were visualized using Super Signal West Dura Extended Duration Substrate (Thermo Scientific, Waltham, MA). Scanning of exposed films were done by CanoScan 9900F (Canon, Oslo, Norway) and signals quantified by the KODAK MI v.4.0.1 software (Kodak, New Haven, CT).

### Transient transfection and plasmid constructs

The NF-κB activity assay was performed as previously described [11]. Briefly, cells were transfected with NF-κB reporter plasmid using electroporation. After overnight incubation, cells were pretreated with inhibitors followed by incubation with 2 μM S100A4 for one hour, harvested and the lysate assayed for luciferase activity using the Luciferase Assay System (Promega, Madison, WI). Kinase dead and wild type constructs of MEKK1 (MEKK1 KD and MEKK1 WT) [22] were purchased from Addgene (Addgene plasmid 12180 and 12181; Cambridge, MA), while NIK KD and WT were kind gifts from Dr. Jacques Piette (Laboratory of Virology and Immunology, University of Liege, Liege, Belgium). MEKK1 and NIK constructs were cotransfected with the NF-κB reporter using the same conditions as described previously [11].

### Real-time RT-PCR

RNA isolation was performed using TRI Reagent^® ^(Ambion/Applied Biosystems, Foster City, CA). Reverse transcription and real-time PCR was performed as previously described [23]. 1 μg total RNA was used for cDNA synthesis, and 1/20 of the reaction mixture employed for each real-time RT-PCR reaction. YARS (tyrosyl-tRNA synthetase) was used as housekeeping gene. Primers used were as previously described [11,24].

### Immunoprecipitation

The IKK complex was immunoprecipitated from unstimulated cells and cells treated with 2 μM S100A4 for the indicated time periods with or without H-7 (30 μM) or staurosporine (0.4 μM). Cells were harvested and cytoplasmic extracts isolated as described by Werner et al [25]. 200 μl cytoplasmic extract was incubated with 1 μg anti-IKKγ (#559675; BD Biosciences, Franklin Lakes, NJ) for 2 hours at 4°C and subsequently with Protein A agarose-conjugated beads (Amersham Biosciences, Uppsala, Sweden) for one hour at 4°C. Beads were washed and collected on Costar Spin-X^® ^centrifuge tube filters (Corning Incorporated, Corning, NY). Proteins were eluted from the beads by adding warm sample buffer (100 mM Tris-HCl pH 6.8, 15% glycerol, 3% SDS, 5% β-mercaptoethanol, 0.1% bromophenol blue and 10 mg/ml DTT), followed by incubation at 95°C for 1 minute and centrifugation at 13 000 rpm for 5 minutes. The immunoprecipitate was separated on 10% NuPAGE^® ^Novex Bis-Tris Gel (Invitrogen, Carlsbad, CA). After blotting the membranes were incubated with anti-phospho-IKKα/β and anti-IKKα.

### IKK activity assay

For IKK activity analyses, the IKK complex from unstimulated and S100A4-treated cells was immunoprecipitated as described above and the kinase reaction performed according to [25]. Briefly, beads containing precipitated IKK complex were incubated in 20 μl kinase buffer containing 20 μM ATP (Fermentas, Vilnius, Lithuania), 10 μCi [^32^P] ATP (Montebello Diagnostics, Oslo, Norway) and 0.5 μg recombinant human IκBα (Cell Sciences, Canton, MA) for 30 minutes at 30°C with or without H-7 (30 μM) or staurosporine (0.4 μM). The beads were pelleted by centrifugation, the reaction mixture was resolved on 10% NuPAGE^® ^Novex Bis-Tris Gels (Invitrogen, Carlsbad, CA) and [^32^P] IκBα was detected by autoradiography. Proteins were eluted from the beads as described above, and the eluate loaded on 10% NuPAGE^® ^Novex Bis-Tris Gels (Invitrogen, Carlsbad, CA), transferred to Immobilon-P membranes (Millipore, Bedford, MA) and immunoblotted using anti-IKKα.

### siRNA transfection

siRNA targeting RAGE was designed by use of the Rational siRNA Design software [26], with minor modifications: antisense: 5'-AACCAACUCUCUCCUGUAU-3', sense: 5'-AUACAGGAGAGAGUUGGUU-3'. *Silencer*^® ^Negative Control #1 siRNA (Ambion/Applied Biosystems, Foster City, CA) was used as negative control. Lipofectamine (Invitrogen, Carlsbad, CA) was used in concentration of 2 μl/ml and cell transfection performed in Opti-MEM (Invitrogen, Carlsbad, CA) according to the manufacturer's procedure. Transfection mixtures contained 50 nM siRNA and the complex was added to 10^6 ^cells seeded in T-25 bottles using reverse transfection. After 24 hours incubation, fresh cell culture medium was added and the cells incubated further for 24 hours. Cells were then stimulated with 2 μM S100A4 for one hour and harvested for protein isolation as described above.

### Statistical analysis

All statistical analyses were performed using two-tailed Student's t-test. Cells treated with S100A4 and H-7/staurosporine were compared to S100A4-stimulated cells without inhibitor. P-values less than 0.05 were considered to be statistically significant.

## Results

### Signal transduction mechanisms involved in S100A4-induced NF-κB activation and expression of target genes

**Figure 1:**
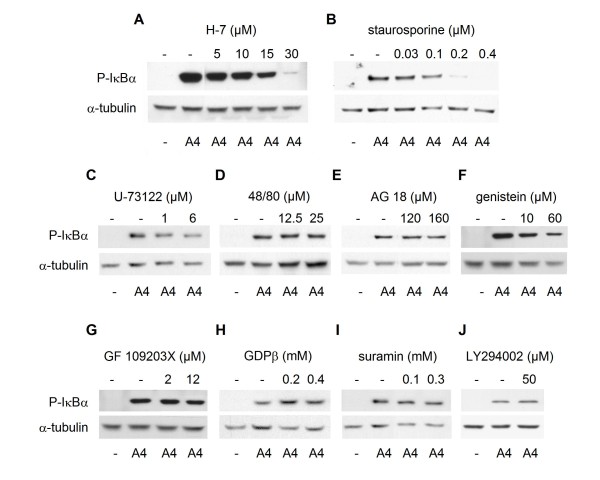
**Dose dependent reduction in phosphorylation of IκBα by Ser/Thr kinase inhibitors**. II-11b cells were pretreated with the inhibitors H-7 (A), staurosporine (B), U-73122 (C) compound 48/80 (D), AG 18 (E), genistein (F), GF 109203X (G), GDPβ (H), suramin (I) or LY294002 (J) using the indicated concentrations. Thereafter, cells were stimulated with 2 μM S100A4 for one hour, harvested and analyzed by immunoblotting for expression of phosphorylated IκBα and α-tubulin. The figures shown are representative of two or three independent experiments.

**Figure 2:**
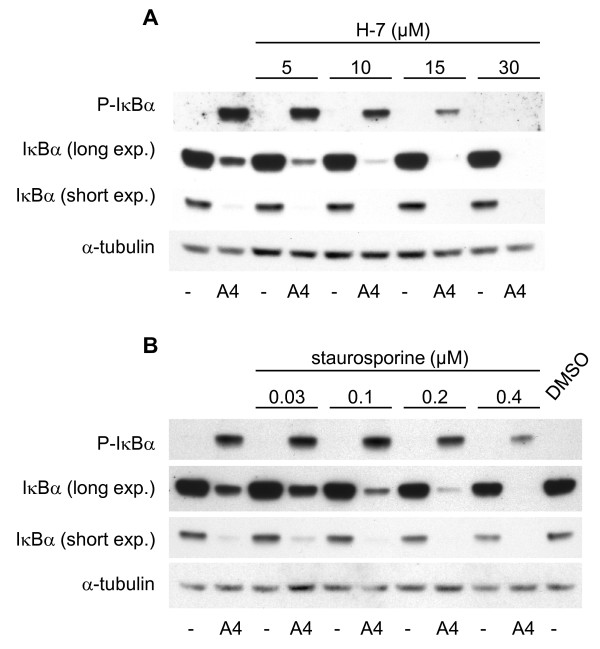
**Dose dependent reduction in phosphorylated IκBα and total IκBα in S100A4-stimulated cells by Ser/Thr kinase inhibitors**. II-11b cells were treated with 2 μM S100A4 and the inhibitors H-7 (A) and staurosporine (B) using the indicated concentrations. After one hour cells were harvested and analyzed by immunoblotting for expression of phosphorylated IκBα, total IκBα and α-tubulin as indicated. For total IκBα both short and long exposure times are shown.

**Figure 3:**
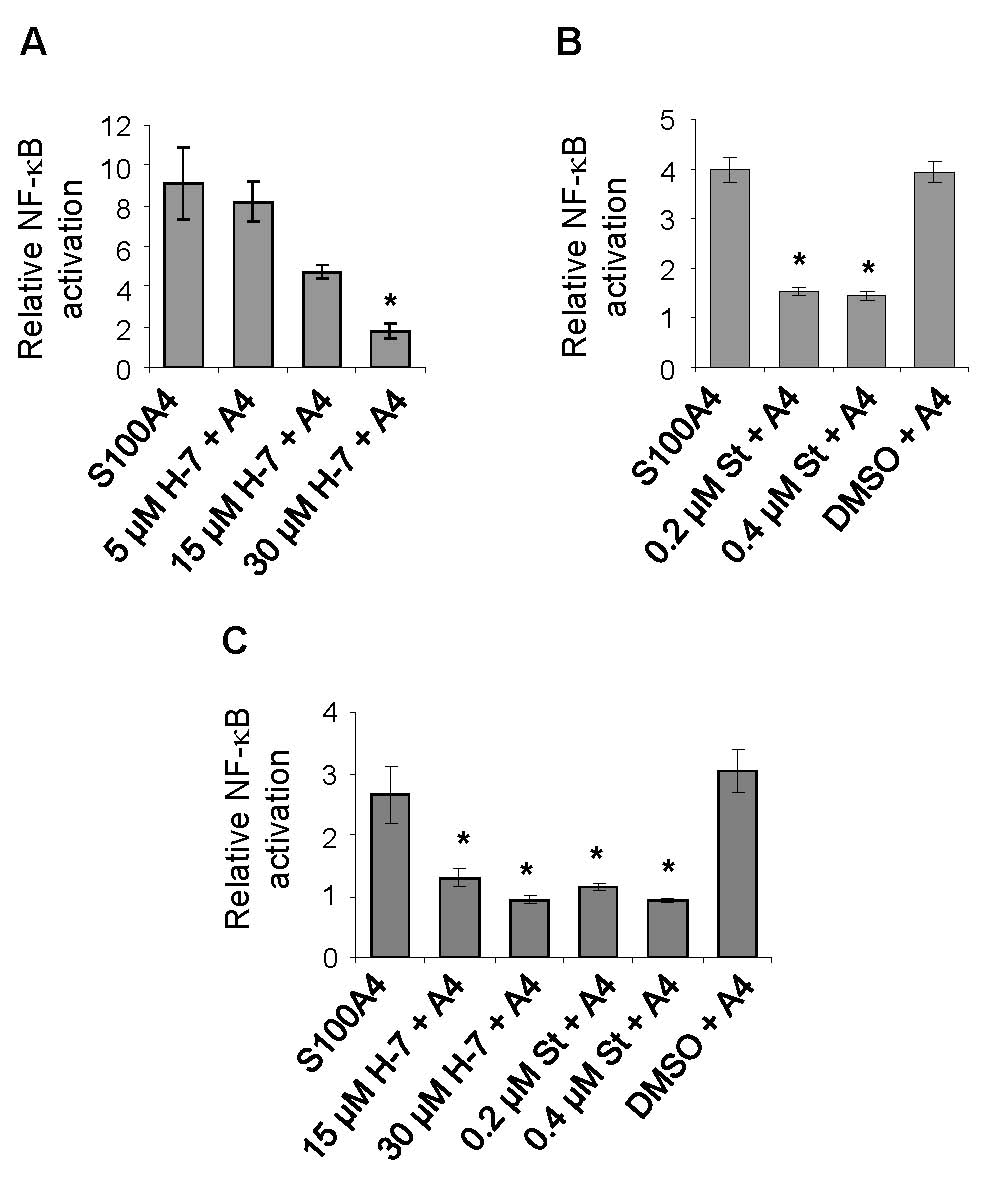
**H-7 and staurosporine inhibit S100A4-induced NF-κB activity**. II-11b (A and B) and KPDX (C) cells transfected with an NF-κB luciferase reporter construct were incubated with H-7 or staurosporine prior to S100A4-stimulation. NF-κB activity is expressed as relative induction upon S100A4 treatment compared to relevant control. Bars represent mean values ± S.E of three independent experiments performed in triplicate or duplicate. St = staurosporine. *, p < 0.05

**Figure 4:**
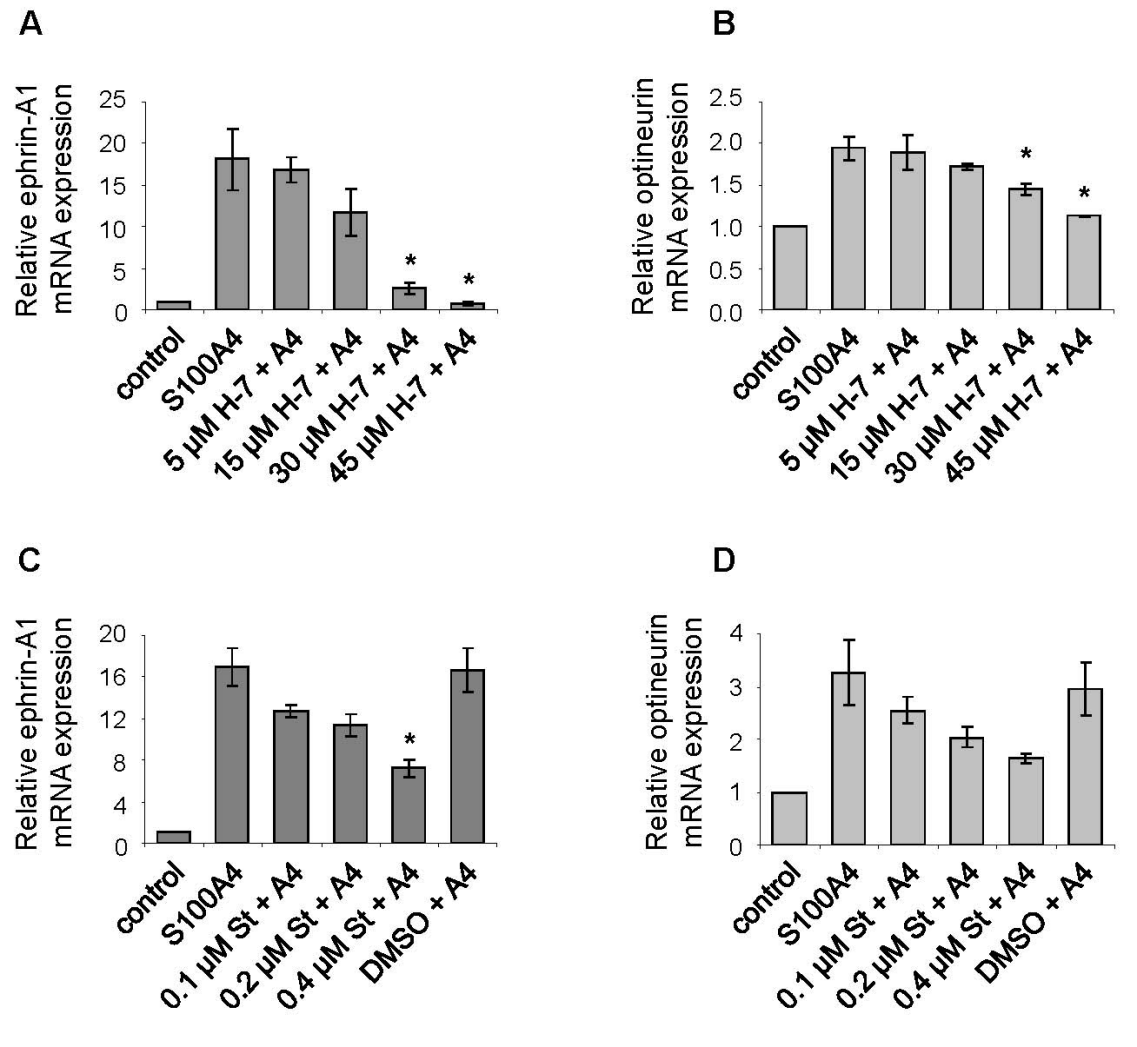
**S100A4 target gene expression is reduced by H-7 and staurosporine**. Real-time RT-PCR demonstrating dose dependent inhibition of ephrin-A1 mRNA (A and C) and optineurin mRNA (B and D) expression in II-11b cells stimulated for 2 hours with 2 μM S100A4 in presence of the indicated concentrations of H-7 (A and B) or staurosporine (C and D). Bars represent mean values ± S.E of three independent experiments. St = staurosporine. *, p < 0.05

We have previously reported that S100A4 stimulates NF-κB activity through the classical activation pathway in the II-11b cell line, by demonstrating increased phosphorylation of IκBα [11]. To investigate the upstream signal transduction mechanisms involved in S100A4-induced NF-κB activation, II-11b cells were treated with inhibitors of various signal transduction pathways: Ser/Thr kinases (H-7 and staurosporine), phospholipase C (U-73122 and compound 48/80), protein tyrosine kinases (AG 18 and genistein), protein kinase C (GF 109203X), G-protein coupled receptors (GDPβ and suramin) and phosphatidylinositol 3-kinases (PI 3-kinases; LY294002). The levels of phosphorylated IκBα were used to measure activity in the NF-κB pathway. The Ser/Thr kinase inhibitors H-7 and staurosporine reduced IκBα phosphorylation levels in a dose dependent manner (Fig. 1A and 1B). A partial inhibitory effect was observed with genistein and compound 48/80 at the highest concentrations, whereas no inhibition was observed with the other signal transduction inhibitors (Fig. 1C-J). Based on these initial experiments, H-7 and staurosporine were chosen for further studies. As previously shown [11], total IκBα expression levels were reduced upon treatment with S100A4 (Fig. 2A and 2B; compare lanes 1 and 2). In S100A4-stimulated cells, increasing concentrations of H-7 or staurosporine resulted in decreased levels of IκBα (Fig. 2A and 2B; compare lanes 4, 6, 8 and 10 with lane 2). IκBα expression was also reduced in cells treated with staurosporine alone. Importantly, H-7 and staurosporine displayed a potent and dose dependent inhibition of S100A4-induced NF-κB activation in a luciferase based activity assay, both using II-11b cells (Fig. 3A and 3B) and the human osteosarcoma cell line KPDX (Fig. 3C). Recently, we demonstrated that S100A4 augmented expression of ephrin-A1 and optineurin in II-11b cells, and that the induction was dependent on NF-κB activity [11]. Addition of H-7 or staurosporine suppressed S100A4-induced expression of these target genes in a dose dependent manner (Fig. 4). Taken together, these findings indicate that Ser/Thr kinases are central players in S100A4-induced NF-κB activation.

### S100A4 induces phosphorylation of IKKα/β

**Figure 5:**
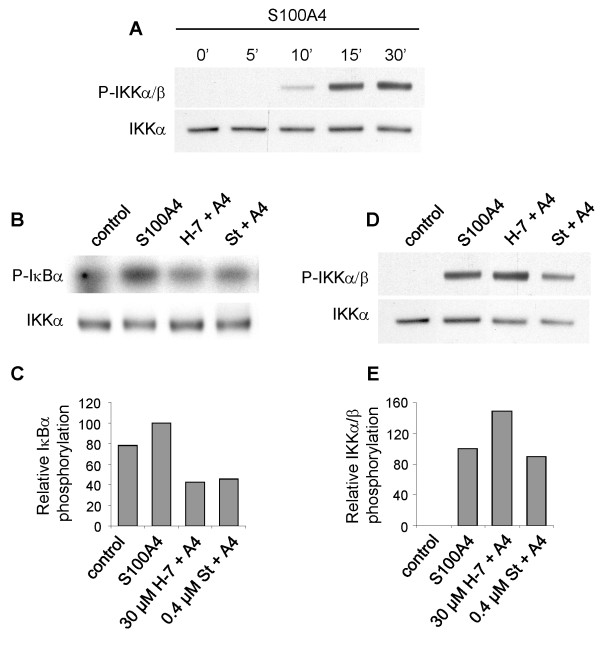
**A. S100A4 induces phosphorylation of IKKα/β**. Western blot of immunoprecipitated IKK complex from II-11b cells treated with 2 μM S100A4 for the indicated time periods and analyzed for expression of phosphorylated IKKα/β. IKKα was used as loading control. B. H-7 and staurosporine inhibit IKK-mediated IκBα phosphorylation in vitro. Upper panel: Kinase assay showing phosphorylation of recombinant IκBα by immunoprecipitated IKK complex from unstimulated II-11b cells or cells stimulated with 2 μM S100A4 for 15 minutes, with or without H-7 (30 μM) or staurosporine (0.4 μM) present in the reaction mixture. Lower panel: Western blot showing IKKα as a loading control. C. Densitometric quantification of the signals shown in Fig. 5B. The bars show IκBα phosphorylation relative to IKKα expression. One of three independent experiments is quantified due to high background in the other experiments. D. Treatment with H-7 or staurosporine did not influence the level of phosphorylated IKKα/β upon S100A4-stimulation for 15 minutes. Immunoprecipitated IKK complex was analyzed by immunoblotting using anti-phospho IKKα/β antibody. IKKα is used as loading control. E. Densitometric quantification of Fig. 5D. Bars show phosphorylated IKKα/β relative to IKKα expression. B and D are representative results of three independent experiments. St = staurosporine.

The IKK complex consists of the two catalytic subunits IKKα and IKKβ and the regulatory subunit IKKγ/NEMO. In the classical NF-κB activation pathway IKKα and IKKβ are activated by phosphorylation of specific serine residues in the activation loop, and is thereby able to induce serine phosphorylation of IκBα [27]. By immunoprecipitating the IKK complex from S100A4-stimulated II-11b cells, and subsequently subject the precipitate to immunoblotting using a phosphospecific IKKα/β antibody, a time dependent induction of IKKα/β phosphorylation was detected (Fig. 5A).

### Inhibitors of Ser/Thr kinases suppress IKK-mediated phosphorylation of IκBα

The observed suppression of S100A4-induced IκBα phosphorylation and NF-κB activation by H-7 and staurosporine could be caused by inhibition of several kinases in the activation pathway. Searching for involved kinases upstream of IκBα, it was of interest to investigate whether H-7 and staurosporine were able to inhibit IKK kinase activity or kinases upstream of IKK. In order to investigate this, the IKK complex was immunoprecipitated from untreated and S100A4-stimulated cells and incubated with recombinant IκBα and radioactive ATP to measure in vitro kinase activity. Fig. 5B and 5C show that S100A4 treatment increased the ability of IKK to phosphorylate IκBα in vitro, while the presence of H-7 or staurosporine reduced IKK-mediated IκBα phosphorylation. To examine whether H-7 or staurosporine affected S100A4-induced activation of the IKK complex, levels of phosphorylated IKKα/β were analyzed in untreated and S100A4-stimulated cells with or without H-7 or staurosporine added to the cell culture medium. A small reduction in phosphorylated IKKα/β was achieved in one of three experiments using staurosporine, whereas H-7 did not suppress the phosphorylation levels (Fig. 5D and 5E). Taken together these results indicate that neither H-7 nor staurosporine inhibits S100A4-induced activation of the IKK complex, while both inhibitors are able to hinder IKK-mediated phosphorylation of IκBα in vitro.

### S100A4-induced NF-κB activation is independent of the Ser/Thr kinases MEKK1, NIK and AKT

**Figure 6:**
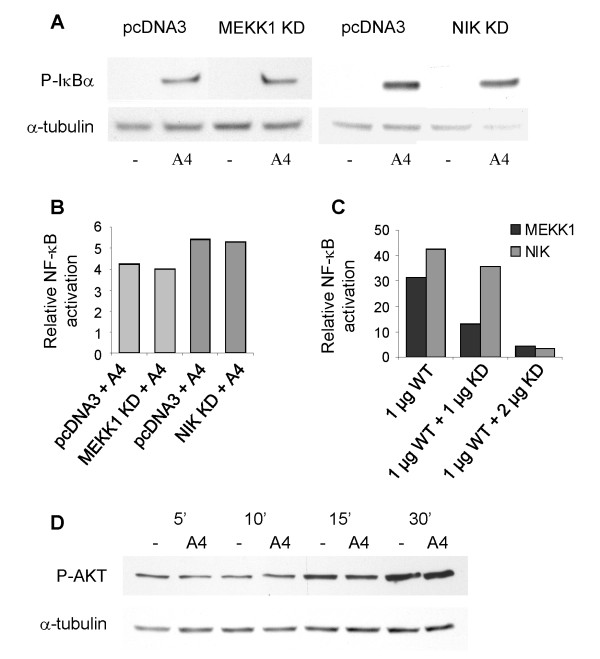
**The Ser/Thr kinases MEKK1, NIK and AKT are not involved in S100A4-mediated activation of the IKK complex**. A. Representative Western blots of II-11b cells transfected with dominant negative constructs of MEKK1 or NIK (2 μg), stimulated with S100A4 for one hour and analyzed for expression of phosphorylated IκBα. B. Cotransfection of II-11b cells with NF-κB luciferase reporter construct and dominant negative MEKK1 or NIK (2 μg) as described in "Methods". Bars show relative induction of NF-κB activity upon S100A4 treatment compared to relevant control. KD = kinase dead. C. Control experiment to evaluate the ability of the dominant negative MEKK1 and NIK constructs to suppress NF-κB activity. Empty vector was used as transfection control and to adjust the amount of plasmid in each transfection. D. Western blot of II-11b cells treated with 2 μM S100A4 for the indicated time periods, analyzed for levels of phosphorylated AKT and α-tubulin. A, B and D are representative results of three independent experiments.

Previously, we demonstrated JNK phosphorylation after S100A4 treatment of II-11b cells [11]. MEKK1 is a possible common upstream kinase responsible for activating both the IKK complex and JNK [28]. It was therefore of interest to examine whether this kinase could be involved in S100A4-induced activation of NF-κB. However, no significant effect was observed on S100A4-induced IκBα phosphorylation or NF-κB activation when dominant negative MEKK1 was overexpressed (Fig. 6A and 6B). It has also been shown that the Ser/Thr kinases NIK and AKT could be involved in phosphorylation and activation of the IKK complex [29,30]. As for MEKK1, dominant negative NIK was not able to inhibit S100A4-mediated IκBα phosphorylation or NF-κB activation (Fig. 6A and 6B). Wild type MEKK1 and NIK was used in experiments to verify that the dominant negative constructs were able to suppress NF-κB activation induced by MEKK1 or NIK (Fig. 6C). Moreover, AKT phosphorylation at serine residue 473 was unaffected by treatment with S100A4 (Fig. 6D). AKT is normally phosphorylated after PI 3-kinase activation, and the finding that LY294002 had no effect on IκBα phosphorylation strengthens the conclusion that AKT is not involved in S100A4-induced IKK activation.

### S100A4-mediated NF-κB activation is RAGE-independent

**Figure 7:**
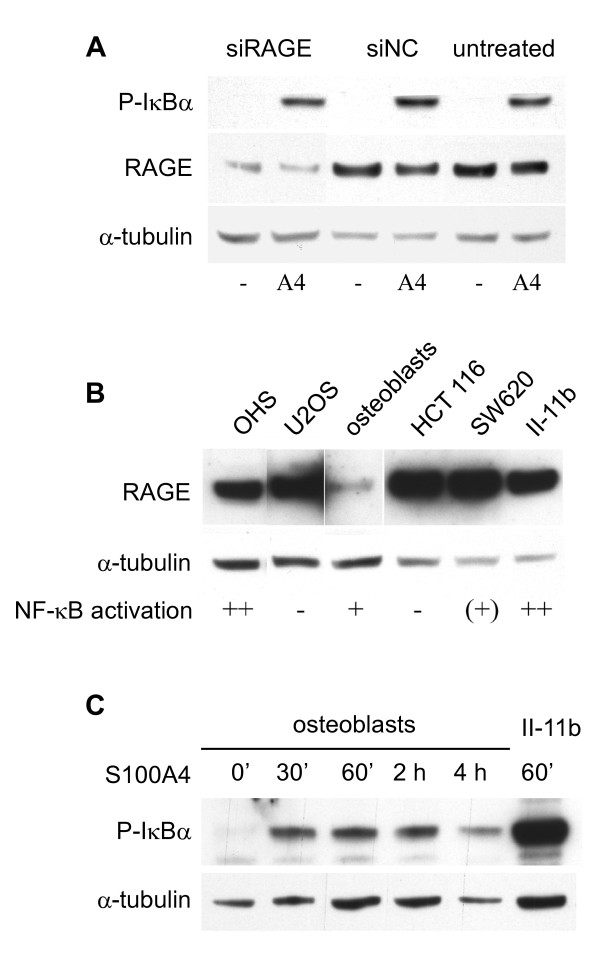
**RAGE-independent NF-κB activation upon S100A4-stimulation of II-11b cells**. A. Western blot showing reduced RAGE protein expression by siRNA transfection. 48 hours after transfection the cells were stimulated with S100A4 for one hour and cell lysates subjected to immunoblotting using anti-phospho-IκBα. The results shown are representative of three independent experiments. siNC = siRNA negative control. B. Western blot showing RAGE expression in a panel of cell lines. NF-κB activation refers to the levels of S100A4-induced NF-κB activation demonstrated in a previous study [11], except for osteoblasts, where it refers to IκBα phosphorylation levels shown in Fig. 7C. C. Western blot showing IκBα phosphorylation in human osteoblasts after S100A4 stimulation for the indicated time periods. α-tubulin was used as loading control.

RAGE has been suggested as receptor for several S100 proteins. In an attempt to investigate the possible role of RAGE in S100A4-induced NF-κB signaling, siRNA molecules targeting RAGE mRNA were utilized. Fig. 7A shows that S100A4 induces phosphorylation of IκBα to the same extent even with RAGE expression levels substantially reduced by siRNA transfection. Furthermore, RAGE expression in a panel of cell lines previously analyzed for NF-κB activation [11] was investigated, and no association between RAGE levels and S100A4-induced NF-κB activation was observed (Fig. 7B). Finally, S100A4-mediated phosphorylation of IκBα was detected in human osteoblasts expressing low levels of RAGE (Fig. 7C). Altogether, these results indicate that RAGE is not involved in S100A4-induced NF-κB activation.

## Discussion

S100A4-induced activation of the transcription factor NF-κB has been reported in several cell systems [6,10,13], but the mechanisms responsible for the enhanced activity are only partly elucidated. We have previously reported that S100A4 activates NF-κB through the classical activation pathway in the II-11b cell line [11], and the present study was initiated to reveal upstream signal transduction mechanisms leading to phosphorylation of IκBα. By using inhibitors of common signal transduction pathways, Ser/Thr kinases were found to be essential for S100A4-induced NF-κB activation. Inhibitors of phospholipase C, protein tyrosine kinases, protein kinase C, G-protein coupled receptors and PI 3-kinases had only a minor or no effect on IκBα phosphorylation in the examined osteosarcoma cell system. S100A4 was for the first time demonstrated to induce IKKα/β phosphorylation. The employed Ser/Thr kinase inhibitors H-7 and staurosporine were able to inhibit the subsequent IKK-mediated phosphorylation of IκBα, NF-κB activation and expression of target genes, whereas the same inhibitors did not affect activation of the IKK complex. RAGE, previously suggested as a receptor for extracellular S100A4 and a well-known activator of NF-κB signaling, was not involved in S100A4-induced NF-κB activation.

Both IκBα and subunits of the IKK complex are phosphorylated on serine residues. It was therefore of interest to examine whether IKK kinase activity or kinases upstream of IKK were suppressed by the added Ser/Thr kinase inhibitors. By utilizing immunoprecipitated IKK complex from S100A4-stimulated cells in an in vitro kinase assay, both inhibitors were demonstrated to reduce IKK-mediated phosphorylation of IκBα. However, the phosphorylation status of the catalytic IKK subunits IKKα and IKKβ were not influenced. The molecular mechanisms of IKK activation have at present not been fully elucidated, but activity is known to depend on phosphorylation of serine residues in the activation loop of IKKα and IKKβ (as detected by the antibody utilized; Fig. 5). This may occur through direct phosphorylation by an upstream kinase, or by trans-autophosphorylation through induced proximity of IKKα/β as a result of IKK multimerization [27]. Because H-7 and the broad spectrum kinase inhibitor staurosporine are able to inhibit IKK-mediated IκBα phosphorylation, one might expect that IKK autophosphorylation also would be suppressed by these inhibitors. In our experiments, IKK phosphorylation was not affected by H-7 and staurosporine, suggesting that an upstream serine kinase could be responsible for the S100A4-mediated IKKα/β phosphorylation. In that event, there are at least three potential explanations for the lack of inhibition by H-7 and staurosporine: (i) the upstream serine kinase is not inhibited by the Ser/Thr kinase inhibitors employed; (ii) the inhibitors were unable to significantly inhibit the upstream kinase at the concentrations and experimental conditions used in our experiments; and (iii) given that signaling components often are functionally redundant, alternative pathways could be activated, masking the inhibitory effect of the particular inhibitor added. Several kinases have been shown to participate in activation of the IKK complex, including the Ser/Thr kinases MEKK1, NIK and AKT [27-31]. Assuming that an upstream serine kinase is involved in S100A4-induced IKK activation, these candidates were further investigated. However, in the II-11b cell line no increase in AKT phosphorylation was observed upon stimulation with S100A4, and dominant negative NIK and MEKK1 had no effect on S100A4-induced NF-κB activation.

The finding that total IκBα levels decreased in S100A4-stimulated cells with increasing concentrations of H-7 and staurosporine was somewhat surprising, and this observation may have at least two explanations. First, IκBα expression is reduced in cells treated with staurosporine alone, indicating that the mechanism is partly S100A4-independent. Second, NF-κB stimulates IκBα transcription as part of a negative feedback mechanism, and the total level of IκBα thus represent the net result of protein degradation and resynthesis. In the II-11b cell line we have previously shown that S100A4-mediated NF-κB activation stimulates transcription of IκBα [11]. Furthermore, the NF-κB transcription complex consists of several proteins, and multiple serine phosphorylations are required for optimal activation [32]. By affecting any of these phosphorylations H-7 and staurosporine may inhibit NF-κB activation, and total IκBα levels may as a consequence decrease upon treatment with S100A4 and the inhibitors compared to S100A4 alone. Nevertheless, the importance and biological relevance of S100A4-induced IκBα phosphorylation is confirmed by previous data showing that S100A4-mediated NF-κB activation is dependent on IκBα phosphorylation at Ser32/36 [11].

The protein tyrosine kinase inhibitor genistein and the phospholipase C inhibitor compound 48/80 displayed a partial inhibition of S100A4-induced IκBα phosphorylation, but we were not able to confirm these results using other inhibitors of the same signaling pathways (AG 18 and U-73122, respectively). Furthermore, inhibitors of protein kinase C, G-protein coupled receptors and PI 3-kinases were unable to affect S100A4-mediated IκBα phosphorylation. Except for PI 3-kinases, the above mentioned mediators have previously been reported involved in S100A4-induced signaling [10,12,15], and the seemingly conflicting results may be explained by cell line specific differences, for instance in expression of cell surface receptors or intracellular signal transduction molecules. As discussed above, functional redundancy may also explain the divergent results.

The effect of S100A4 on IKK phosphorylation was detected as early as after 10 minutes, indicating receptor-mediated transduction of the signal from the extracellular environment to intracellular effector molecules. Many S100 proteins have been found to transduce their effects through RAGE, but RAGE-independent effects have been observed both for S100A4 and other S100 proteins [14]. NF-κB activation is a well-known downstream event of RAGE signaling, and NF-κB activation by S100A4 [10], S100A1 [33], S100A8/A9 [34], S100A12 [35], S100B [33] and S100P [36] has been shown to be RAGE-dependent in certain cell systems. Therefore, we examined the involvement of RAGE in S100A4-induced NF-κB activation in II-11b cells. Using siRNA molecules targeting RAGE mRNA, expression was substantially reduced without observing any effect on S100A4-stimulated IκBα phosphorylation. The exact protein expression levels of RAGE necessary to maintain downstream signal transduction is not known, but the observed reduction in RAGE expression was more pronounced than in other studies demonstrating RAGE-dependent effects [34], suggesting that the observed S100A4-mediated activation of NF-κB is RAGE-independent. Accordingly, S100A4-induced neurite outgrowth occurs through RAGE-independent mechanisms [15], and extracellular S100A4 stimulates motility and activates NF-κB in cells that do not express RAGE mRNA [6,7].

Signaling through RAGE is clearly responsible for biological effects induced by extracellular S100A4 in certain cell systems, but other cell surface molecules have also been suggested to be involved in S100A4 signaling. Neurite outgrowth mediated by S100A4 was partly dependent on interaction with heparan sulfate proteoglycans at the cell surface [15], and other S100 proteins also bind heparan sulfate moieties [37]. In the II-11b cell line treatment with heparin had no effect on S100A4-induced NF-κB activation (results not shown), indicating that S100A4-induced NF-κB signaling is not dependent on interaction with glycosaminoglycans at the cell surface. On endothelial cells, S100A4 interacts with annexin II, which acts as a coreceptor governing the assembly of S100A4, plasminogen and its activators [8]. However, annexin II is not known to propagate intracellular signals upon binding to S100A4. Altogether, our findings suggest that a so far unidentified cell surface receptor mediates S100A4-induced NF-κB activation.

## Conclusions

Extracellular signals enhancing tumor cells metastatic capacity might be attractive candidates for therapeutic intervention. One such candidate is the metastasis-promoting protein S100A4. In the present study we used a human osteosarcoma cell line to demonstrate that extracellular S100A4 activates the IKK complex and induces NF-κB activity independent of the postulated S100 receptor RAGE. Further studies to identify possible S100A4 specific receptor molecule(s) and induced downstream signaling pathway(s) may identify targets that can be utilized in anti-metastatic therapy.

## Competing interests

The authors declare that they have no competing interests.

## Authors' contributions

IG carried out all the laboratory experiments and drafted the manuscript. GMM and KB conceived and designed the study. All authors read and approved the final manuscript.

## Pre-publication history

The pre-publication history for this paper can be accessed here:

http://www.biomedcentral.com/1471-2407/10/241/prepub
